# Leveraging Large and Diverse Biobanks to Evaluate Gene–Disease Associations in Hypertrophic Cardiomyopathy †

**DOI:** 10.3390/jpm16030171

**Published:** 2026-03-21

**Authors:** Saif F. Dababneh, Kevin Ong, Darwin Yeung, Nathaniel M. Hawkins, Andrew Krahn, Zachary Laksman, Rafik Tadros, Thomas M. Roston

**Affiliations:** 1 Department of Cellular and Physiological Sciences, University of British Columbia, Vancouver, BC V6T 1Z4, Canada; 2 Cellular and Regenerative Medicine Centre, BC Children’s Hospital Research Institute, Vancouver, BC V5Z 4H4, Canada; 3 Division of Cardiology, Centre for Cardiovascular Innovation, The University of British Columbia, Vancouver, BC V6T 1Z4, Canada; 4 School of Biomedical Engineering, University of British Columbia, Vancouver, BC V6T 1Z4, Canada; 5 Electrophysiology Service and Cardiovascular Genetics Centre, Montreal Heart Institute, Université de Montréal, Montreal, QC H2V 0B3, Canada

**Keywords:** HCM, ClinGen, biobanks

## Abstract

**Background:** Hypertrophic cardiomyopathy (HCM) is a common inherited disease and a leading known cause of sudden cardiac arrest in young adults and athletes. While genetic testing has advanced rapidly in the past decade, the yield of genetic testing remains low. The Clinical Genome Resource (ClinGen) initiative has become a leading resource for defining the clinical relevance of genetic variants with expert groups focusing on evaluating the strength of evidence for each HCM implicated gene. With the rise of large biobanks and population databases, genetic discovery has been significantly advanced. However, whether these databases can be used to validate gene–disease associations curated by ClinGen and provide evidence for novel gene–disease associations remains unclear. **Objectives:** Here, we utilized a publicly available database containing 748,879 individuals across three large biobanks (All of Us, UK biobank, Mass General Brigham biobank). **Methods:** We tested the association of rare coding variants in each gene in the HCM ClinGen panel with HCM. In total, 38 genes were tested, and Bonferroni correction was applied accordingly. **Results:** Of the 12 genes with definitive evidence for HCM (e.g., *MYBPC3*, *MYH7*, *TNNT2*, *ALPK3*), 8 (67%) demonstrated nominally significant association with HCM on a population level, and 5 (42%) remained significant after Bonferroni correction, further supporting the validity of these genes in HCM panels. Several definitive genes which are much less commonly affected in HCM (*CSRP3*, *MYL3*, *ACTC1*, *TPM1*, *FHOD3*, *MYL2*, and *TNNC1*) did not pass our Bonferroni corrected-significance threshold, but all had positively associated effect sizes with HCM. No genes deemed to have moderate or limited evidence had any significant associations with HCM even before Bonferroni correction. **Conclusions:** Altogether, we show that large biobanks and population databases generally recapitulate established gene–disease associations for HCM and support the ClinGen group’s gene curations. The utilization of such publicly accessible databases represents an additional tool for assessing gene validity in monogenic cardiac disorders with an established phenotype, although it may have limited sensitivity and should not be solely relied on.

## 1. Introduction

Hypertrophic cardiomyopathy (HCM) is a common heritable cardiac disease that can lead to left ventricular outflow tract obstruction, arrhythmias, heart failure and sudden death. Clinically, HCM is characterized by asymmetrical left ventricular hypertrophy, myocyte disarray, and myocardial fibrosis, which subsequently lead to diastolic dysfunction, heart failure, and malignant ventricular arrhythmias [1,2]. HCM is typically an autosomal dominant, Mendelian genetic disorder caused by pathogenic genetic variants usually in sarcomeric proteins, including *MYH7*, *MYBPC3*, and *TNNT2*, although some non-sarcomeric proteins have also been implicated, such as *ALPK3*, *FHOD3*, *FLNC*, and *PLN* [3,4].

While genetic testing technologies have advanced rapidly in the past decade, the yield of genetic testing in HCM remains modest. Pathogenic or likely pathogenic variants are identified in only 30-40% of affected individuals, with higher yields in familial cases, leaving a substantial proportion of patients without an identifiable monogenic cause [5]. Moreover, many individuals harbor variants of uncertain significance (VUSs), particularly in genes with limited or emerging evidence for disease association, rendering clinical interpretation and genetic counseling more challenging [6]. The use of pre-clinical models and human stem cell-derived cardiomyocytes has helped shed light on the pathogenicity of some variants, but the number VUSs vastly supersedes our ability to functionally characterize each variant [7]. Unrecognized genes, intermediate effect variants, and polygenic risk likely account for the >50% of patients not harboring a clear pathogenic variant in a well-established HCM gene (i.e., so-called “gene negative” HCM) [8,9,10,11,12]. This is notably compounded in ethnically diverse populations, as population-level genomic control data are lacking [13,14,15]. However, some uncertainty continues to exist related to the genetic architecture of HCM, particularly for more recently identified genes.

The uncertainty around gene–disease association in HCM, and many other inherited cardiac disorders, has been a central problem which has largely been driven by small observational studies supported by weak evidence. Unfortunately, this has led to many genes being included on clinical gene sequencing panels which lack sufficient evidence for disease causation. To address this widespread problem, the Clinical Genome Resource (ClinGen) initiative has become a leading resource for defining the clinical relevance of genes implicated in HCM with expert groups focusing on evaluating the strength of evidence for each HCM implicated gene [4]. At present, this process involves extensive evaluation of the published literature. However, the rise of large biobanks and population-level databases has markedly advanced our understanding of genetic disease across general and ethnically diverse populations. Whether these databases can be used to validate gene–disease associations curated by ClinGen and provide evidence for novel gene–disease associations remains unclear. In this paper, we sought to explore the utility of large, publicly available biobanks and population databases in validating ClinGen’s HCM curation by testing the association of each gene in the curated list with HCM incidence. This was undertaken using a publicly available dataset comprising a diverse population of 748,879 individuals who have undergone whole exome or genome sequencing.

## 2. Materials and Methods

### 2.1. Biobanks and Databases

The UK Biobank (https://www.ukbiobank.ac.uk/, last accessed 15 December 2025) is a large-scale, prospective population-based cohort comprising around 500,000 participants from across the United Kingdom. Recruitment began in 2006 and targeted individuals aged 40–69 years. Participants provided extensive health-related information, biological samples, and genetic data, including whole-exome and whole-genome sequencing. The Mass General Brigham (MGB) Biobank (https://www.massgeneralbrigham.org/en/research-and-innovation/participate-in-research/biobank, last accessed 15 December 2025) is a hospital-based cohort of over 160,000 patients aged 18 years and older within the Mass General Brigham health system. This biobank links electronic health records with whole-exome sequencing and biological samples. The All of Us Research Program (https://www.researchallofus.org, last accessed 15 December 2025) is an ongoing, prospective, population-based biobank aiming to enroll over one million participants from diverse and historically underrepresented populations in the United States. Participants, recruited from age 18 and older in the first phase, provide electronic health records, biological samples, and genetic data, including whole-genome sequencing. All three biobanks received appropriate ethics approval, and all participants provided informed consent prior to enrollment.

We utilized a publicly available, online browser-based, phenome-wide association study (PheWAS) database which tested the association of rare coding variants with multiple phenotypes in 748,879 individuals across these three large biobanks (All of Us, UK biobank, Mass General Brigham biobank), 155,236 of which are of non-European ancestry [16]. This dataset was limited to gene-level analysis and does not provide data on specific variants. The analysis was specified to rare variants (minor allele frequency <0.001) and the one ICD-10 phenocode available for HCM (“I42.2—Other hypertrophic cardiomyopathy”) in this PheWAS database. Hence, ICD-10 code “I42.1—Obstructive hypertrophic cardiomyopathy” was not tested here. Six burden testing masks were tested in this PheWAS: loss-of-function (LOF) and minor allele frequency (MAF) < 0.001, LOF + missense 0.8 and MAF < 0.001, LOF + missense 0.5 and MAF < 0.001, LOF + missense 0.5 and MAF < 0.00001, missense 0.5 and MAF < 0.00001, and missense 0.2 and MAF < 0.00001). The missense score is derived from a combination of multiple computation tools with the ratio representing the number of tools predicting pathogenicity over the total number of tools tested [16]. Variants were annotated by the authors using dbNSFP and loss-of-function transcript effect estimator (LOFTEE). The missense score integrates values from 30 computational prediction tools within dbNSFP, with the ratio representing the number of tools predicting the missense variant to be damaging divided by number of tools used, as described in detail by Jurgens et al. [16]. Other details of the phenome-wide association study, including all the genetic epidemiology methods, are detailed by Jurgens et al. [16].

### 2.2. ClinGen HCM Gene Curation

We evaluated 38 genes with ClinGen gene–disease validity classifications for non-syndromic HCM (https://search.clinicalgenome.org/kb/conditions/MONDO:0005045, last accessed 15 December 2025) [3,4]. The list of genes under each classification are as follows. Definitive: *MYBPC3*, *MYH7*, *ALPK3*, *TNNT2*, *TNNI3*, *CSRP3*, *MYL3*, *ACTC1*, *TPM1*, *MYL2*, *TNNC1*, *FHOD3.* Moderate: *TRIM63*, *KLHL24*, *JPH2*, *MT-TI*. Limited: *RBM20*, *NEXN*, *TTN*, *OBSCN*, *TMPO*, *KLF10*, *PDLIM3*, *RYR2*, *RPS6KB1*. Disputed: *MYOM1*, *DSP*, *ANKRD1*, *TCAP*, *VCL*, *KCNQ1*, *MYLK2*, *CALR3*, *MYH6*, *CACNB2*, *MYOZ2*, *CASQ2*, *MYPN*. No known disease relationship: *TNNC2*. We did not test for syndromic HCM genes not in the ClinGen list (e.g., *FLNC*, *PRKAG2*, *LAMP2*, *TTR*, *GLA*).

### 2.3. Statistical Analysis

A two-sided *p* value < 0.05 was considered nominally significant. We specifically used the cauchy *p* value provided by the PheWAS, which integrates the *p* values from all six rare burden testing masks into one. However, we also applied a Bonferroni correction to account for multiple-gene testing for 38 genes, whereby the adjusted *p* value threshold (*p*_adj_) was <0.00132 (0.05/38 genes).

## 3. Results

**Figure 1:**
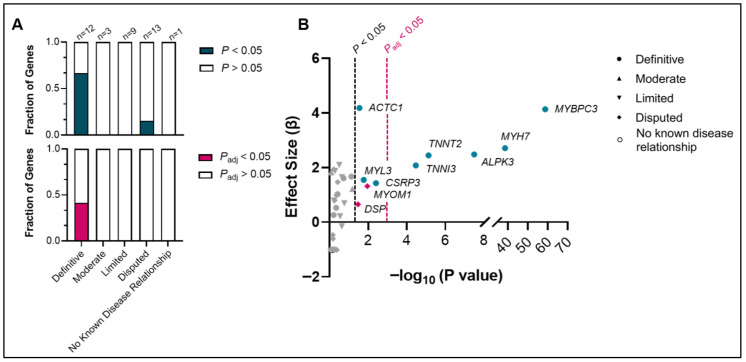
Gene–disease associations with HCM in large biobanks and databases. (**A**) Fraction of genes with significant associations with HCM stratified by their ClinGen classification. (**B**) Plot showing the relationship between the magnitude and significance of each gene’s association with HCM. Only genes with significant associations are highlighted and labeled. Each point’s shape represents its ClinGen’s HCM gene–disease validity classification. Nominal significance (i.e., *p* value without adjustment for multiple-gene testing) was defined as *p* < 0.05, while statistical significance with Bonferroni correction was defined as *p*_adj_ < 0.05. Both thresholds are shown for comparison.

We tested the association of each gene in the HCM ClinGen panel with HCM, which was stratified by the gene’s ClinGen evidence classification (definitive, moderate, limited, disputed, no known disease relationship). Of the 12 genes with definitive evidence for HCM (e.g., *MYBPC3*, *MYH7*, *TNNT2*, *ALPK3*, *TNNI3*, *CSRP3*), 8 (67%) demonstrated nominally significant associations (i.e., *p* < 0.05) with HCM on a population level, and 5 of those (42%) remained significant (i.e., *p*_adj_ < 0.05) with Bonferroni correction (*MYBPC3*, *MYH7*, *ALPK3*, *TNNT2*, *TNNI3*), further supporting the validity of these genes in HCM panels (Figure 1A,B).

Three definitive HCM genes (*CSRP3*, *MYL3*, and *ACTC1*) were nominally significant but did not reach significance after Bonferroni correction. Four definitive HCM genes (*TPM1*, *FHOD3*, *MYL2*, and *TNNC1*) did not reach nominal significance, which was possibly due to being ultra-rare causes of HCM. The allele frequencies, effect sizes, and *p* values for the 12 definitive genes are provided in Supplementary Table S1.

No genes deemed to have moderate (n = 3) (e.g., *KLHL24*) or limited (n = 9) (e.g., *NEXN*) evidence were significantly associated with HCM. Interestingly, within the 13 disputed genes, two (15%) genes (*MYOM1*, *DSP*) were nominally significant but had notably lower effect sizes and greater *p* values compared to definitive genes and did not reach the adjusted *p* value cutoff (Figure 1A,B).

## 4. Discussion

In this paper, we leveraged a publicly available dataset which analyzed three large biobanks comprising nearly 750,000 individuals to assess gene–disease associations for the HCM-implicated genes in accordance with their ClinGen classification. Our findings demonstrate that large population-based datasets can generally recapitulate established gene–disease associations for HCM, especially for genes with variants that are commonly implicated in HCM, providing independent support for the clinical validity of genes classified as definitive by ClinGen. This also suggests that future gene–disease validation initiatives could be well served by integrating these data sources for a variety of well-defined monogenic human diseases.

Several findings are of particular interest. In HCM, eight of the 12 genes classified as “definitive” by ClinGen, including *MYBPC3*, *MYH7*, *TNNT2*, and *ALPK3*, were nominally significantly associated with HCM at the population level, five of which remained significant after adjusting for multiple-gene testing. These results reinforce the inclusion of these genes in HCM genetic testing panels and suggest that population-scale sequencing data can serve as a valuable tool for validating gene–disease relationships. While most of these genes have been well established, particularly sarcomeric genes, our understanding of *ALPK3* and its robust role in HCM pathogenesis is relatively newer. It was previously thought that *ALPK3*, which encodes a nuclear pseudokinase critical for sarcomeric and protein quality control proteins [17,18], can only cause HCM in an autosomal recessive or compound heterozygous manner [19]. Here, five patients had homozygous *ALPK3* truncating variants. However, there is increasing evidence that heterozygous *ALPK3* truncating variants could also lead to a later-onset HCM phenotype in an autosomal dominant fashion [20,21]. Our paper further supports evidence for *ALPK3* causing autosomal dominant HCM given its robust association with HCM in a population-based database where homozygous carriers are likely to be much less prevalent, although this needs validation and an analysis of individual variants, as compound heterozygotes are possible. Efforts should be made to ensure that those families historically affected by *ALPK3* variants undergo cascade screening.

The use of large-scale biobanks may also provide valuable insights HCM by enabling longitudinal genotype–phenotype correlations, including relationships between the variant type, age of onset, and clinical outcomes, thereby refining risk stratification and prognostication. For instance, one UK biobank study identified the prevalence of rare variants in HCM-associated sarcomeric genes to be 2.9% in the general population, and it compared the lifetime outcomes of sarcomere-positive versus sarcomere-negative HCM participants [22].

There were seven genes classified by ClinGen as “definitive” that were either nominally significant but did not reach statistical significance after Bonferroni correction (*CSRP3*, *MYL3*, and *ACTC1*) or never reached nominal significance (*TPM1*, *FHOD3*, *MYL2*, and *TNNC1*). Pathogenic variants in these genes are considered ultra-rare in HCM [23,24,25]. For instance, *MYL2* and *MYL3* variants are collectively responsible for <1% of HCM cases [1,2]. Hence, the lack of statistical significance most likely reflects limited power for detecting associations with extremely low-frequency pathogenic variants. The growth of population datasets may eventually be able to provide more definitive results for ultra-rare conditions. At this time, the lack of association between these genes and HCM certainly does not invalidate the conclusions made by the ClinGen consortium which integrate gene-based association analyses as well as other levels of evidence including human genetics and experimental data.

Interestingly, within the set of disputed genes, two genes (*MYOM1* and *DSP*) reached nominal significance but not statistical, Bonferroni-corrected significance. *MYOM1* and *DSP* both encode proteins important for sarcomere and cytoskeletal integrity [26,27], and *DSP* is an established causal gene in arrhythmogenic cardiomyopathy [28]. However, the observed effect sizes were notably lower and *p* values were higher compared to definitive genes, suggesting a weaker association, if any, with HCM. There are several potential explanations. One explanation could be the lack of robust standardization in ICD-10 coding. There is also the potential phenotypic heterogeneity in carriers of *MYOM1* or *DSP* which may be underappreciated, such as the presence of mild and nonspecific hypertrophy despite a more dominant non-HCM cardiac phenotype. These associations may also be due to chance alone given their nominal significance and not passing the threshold after adjusting for multiple-gene testing.

Other limitations in our analysis are as follows. First, there was only one ICD-10 code for HCM tested in the PheWAS we used, and ‘I42.1—Obstructive Hypertrophic Cardiomyopathy’ was not tested, limiting our ability to specifically test for associations with the obstructive HCM phenotype. A potential misclassification or incomplete capture of HCM diagnoses in electronic health records may have attenuated associations. Second, our analysis was limited to gene-level variant burden testing and we did not have access to individual variant data, preventing any assessment of heterozygous versus homozygous contributions or evaluation of specific pathogenic variants. Third, genes with ultrarare variants remain underpowered for detection, as observed with *TPM1*, *FHOD3*, *MYL2*, and *TNNC1*. Lastly, we did not test the associations for syndromic HCM genes not included in the ClinGen HCM list (e.g., *FLNC*, *PRKAG2*, *LAMP2*, *TTR*, *GLA*).

## 5. Conclusions

Altogether, our analysis demonstrates that large biobanks and population databases generally recapitulate established gene–disease associations for HCM, particularly amongst more common HCM genotypes, supporting the ClinGen group’s gene curations. The utilization of publicly accessible databases represents a promising tool for assessing gene validity but may have limited additional value in HCM by itself given that HCM’s monogenic basis is already well defined. Nevertheless, this approach may also be useful in validating novel candidate HCM genes across diverse populations. We propose that future gene validity initiatives could leverage existing population level databases in a similar manner.

## Data Availability

The original data presented in this paper are openly available in https://hugeamp.org:8000/research.html?pageid=600_traits_app (last accessed 15 December 2025), which has been published by Jurgens et al. [15].

## Supplementary Materials

The following supporting information can be downloaded at: https://www.mdpi.com/article/10.3390/jpm16030171/s1, Table S1: Minor Allele Frequencies of Definitive HCM Genes.
